# Differentiation between spinal multiple myeloma and metastases originated from lung using multi-view attention-guided network

**DOI:** 10.3389/fonc.2022.981769

**Published:** 2022-09-08

**Authors:** Kaili Chen, Jiashi Cao, Xin Zhang, Xiang Wang, Xiangyu Zhao, Qingchu Li, Song Chen, Peng Wang, Tielong Liu, Juan Du, Shiyuan Liu, Lichi Zhang

**Affiliations:** ^1^ Department of Hematology, Myeloma & Lymphoma Center, Shanghai Changzheng Hospital, Changzheng Hospital of the Naval Medical University, Huangpu, China; ^2^ Department of Orthopedics, No. 455 Hospital of Chinese People’s Liberation Army, Shanghai 455 Hospital, Navy Medical University, Shanghai, China; ^3^ Department of Orthopaedic Oncology, Spine Tumor Center, Shanghai Changzheng Hospital, Changzheng Hospital of the Navy Medical University, Huangpu, China; ^4^ Institute for Medical Image Technology, School of Biomedical Engineering, Shanghai Jiao Tong University, Shanghai, China; ^5^ Department of Radiology, Changzheng Hospital, Shanghai Changzheng Hospital, Navy Medical University, Huangpu, China

**Keywords:** multiple myeloma (MM), spinal metastases, lung cancer, deep learning, attention guidance strategy, radiomics

## Abstract

**Purpose:**

Multiple myeloma (MM) and metastasis originated are the two common malignancy diseases in the spine. They usually show similar imaging patterns and are highly demanded to differentiate for precision diagnosis and treatment planning. The objective of this study is therefore to construct a novel deep-learning-based method for effective differentiation of two diseases, with the comparative study of traditional radiomics analysis.

**Methods:**

We retrospectively enrolled a total of 217 patients with 269 lesions, who were diagnosed with spinal MM (79 cases, 81 lesions) or spinal metastases originated from lung cancer (138 cases, 188 lesions) confirmed by postoperative pathology. Magnetic resonance imaging (MRI) sequences of all patients were collected and reviewed. A novel deep learning model of the Multi-view Attention-Guided Network (MAGN) was constructed based on contrast-enhanced T1WI (CET1) sequences. The constructed model extracts features from three views (sagittal, coronal and axial) and fused them for a more comprehensive differentiation analysis, and the attention guidance strategy is adopted for improving the classification performance, and increasing the interpretability of the method. The diagnostic efficiency among MAGN, radiomics model and the radiologist assessment were compared by the area under the receiver operating characteristic curve (AUC).

**Results:**

Ablation studies were conducted to demonstrate the validity of multi-view fusion and attention guidance strategies: It has shown that the diagnostic model using multi-view fusion achieved higher diagnostic performance [ACC (0.79), AUC (0.77) and F1-score (0.67)] than those using single-view (sagittal, axial and coronal) images. Besides, MAGN incorporating attention guidance strategy further boosted performance as the ACC, AUC and F1-scores reached 0.81, 0.78 and 0.71, respectively. In addition, the MAGN outperforms the radiomics methods and radiologist assessment. The highest ACC, AUC and F1-score for the latter two methods were 0.71, 0.76 & 0.54, and 0.69, 0.71, & 0.65, respectively.

**Conclusions:**

The proposed MAGN can achieve satisfactory performance in differentiating spinal MM between metastases originating from lung cancer, which also outperforms the radiomics method and radiologist assessment.

## Introduction

Multiple myeloma (MM) and metastases are two common malignant tumors involving spine. MM is the second most common hematological malignancy with an incidence of 4.6-6 per 10,0000 per year (1). Almost 70% of all bone metastases occur in the spinal column, which is third most commonly affected by metastases (2). The occurrences of both diseases are on the rise because of an increase in overall cancer survival and the ageing population (3, 4). Although spinal MM and metastases are two different tumoral entities with completely different management strategies, both can be presented as single or multiple lesions in the spine, leading to diverse and atypical clinical manifestations, including bone pain, neurologic deficits, and even paralysis with compression of the spinal cord. In addition, spinal MM and metastasis have shown similar imaging patterns on conventional imaging methods, appearing as lytic changes in x-ray and CT images, and overlaps of MR imaging patterns (5). These factors create great difficulties in the differential diagnosis of the two diseases especially for those situations where some MM are non-secretory or hypo-secretory and patients have spinal metastases of unknown origin (SMUO) (6, 7). Specific chemotherapy and radiation therapy are currently the two main options for the treatment of patients with MM, yet spinal metastases are treated relatively conservatively with further treatment strategies focusing on primary tumors (1, 4, 5, 8, 9). Since there are significant differences in the treatment planning and prognosis of the two diseases, early diagnose and differential diagnose plays a key role in the individual treatment for patients with spinal MM and metastases.

Conventional imaging methods have been the mainstream approach in imaging diagnosis of spinal tumors, including X ray, computed tomography (CT), and MRI. MRI is the most widely used imaging modality for diagnosing tumors in the spine. Compared with CT and X ray, MRI has the advantage of marrow infiltration assessment (10). However, many studies have revealed that it is often difficult to distinguish spinal MM from metastases by standard MRI alone, especially for patients with multiple vertebral focal osteolytic lesions and other atypical manifestation (11, 12). Many studies pointed out that advanced MRI including contrast-enhanced (DCE) MRI and diffusion-weighted imaging (DWI) MRI could help differential diagnosis in the two diseases (5, 11–13). But relevant studies are still limited and their findings require further investigation. The 18F-Fluorodeoxyglucose (18F-FDG) positron emission tomography (PET) and computed tomography (18F-FDG PET/CT) is recommended as one the best imaging methods in detecting MM and metastases (1, 5, 14). However, it comes with disadvantages such as high exposure to radiation and expensive costs for patients. More importantly, identification of lesions by these methods mentioned above relies on subjective visual assessment and diagnostic experience, which is undoubtedly a great challenge for younger radiologists.

Recently, radiomics and deep learning(DL) technology have been fast developed, and also demonstrates outstanding potential in many computer-aided diagnosis applications, which serve as new tools to transcend subjective visual assessment and provide more objective evaluation for diseases. Several studies successfully applied radiomics analysis for differentiating spinal MM from metastases and yielded satisfied outcomes (5, 14, 15). However, other studies revealed that radiomics has some limitations, including poor repeatability as well as reliability, and time-consuming and cumbersome workflows (16). DL can automatically extract comprehensive features based on the specific classification tasks and has been reported with better performance for differential diagnosis in spinal metastases compared with radiomics (6). Nevertheless, there remains no study to date focusing on differentiating spinal MM and metastasis based on the DL model, not alone comparison between the DL and radiomics.

This study aims to develop a novel deep-learning-based method to differentiate spinal MM from metastases using contrast-enhanced T1WI (CET1) sequences. Herein, we chose metastases originating from the lung cancer as the group of metastases, which are the most common type of spinal metastases (7, 17). Moreover, we designed two strategies in the method to guarantee its effectiveness: 1) we employed the MR images from three different views (sagittal, axial and coronal), and designed the multi-view feature extraction and fusion strategies to conduct the differentiation process more comprehensively; 2) we incorporated the attention-guidance module in the model framework, which could provide prior guidance for the trained model to focus more on the lesion regions, aiming at further improving its classification performance. Besides, such attention guidance could reduce the need for numerous training samples when constructing the deep learning model. We finally validated the diagnostic performance of our method using the collected dataset, which was also compared with the radiomics analysis and radiologist assessment.

## Materials and methods

### Participants

**Figure 1:**
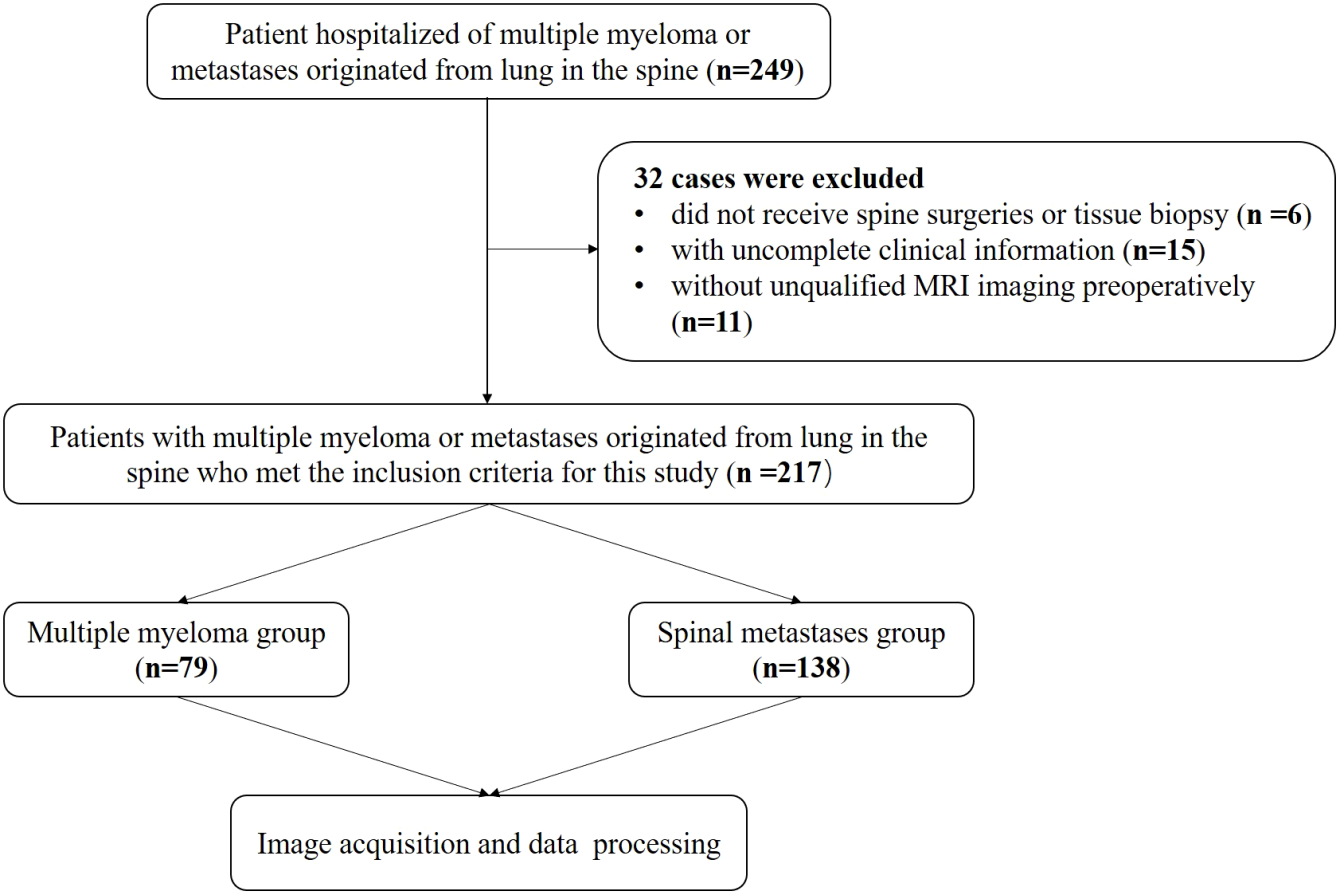
The flowchart of patients’ inclusion and exclusion details.

This retrospective study was approved by the Ethics Committee of Changzheng Hospital of the Navy Medical University, and the informed consent was waived. From January 2014 to December 2020, we retrospected clinically and MRI information of patients, who received spine surgery or needle biopsy and were diagnosed with MM or spinal metastases originating from the lung cancer. The inclusion criteria were (1): patients diagnosed with spinal MM and metastasis confirmed by histological or cellular pathology; (2) patients examined with preoperative MRI in our hospital to ensure qualified MRI images; and (3) patients with complete clinical information. Exclusion criterion: (1) patients diagnosed with other spinal tumors confirmed by histological or cellular pathology; (2) Patients who did not receive preoperative MRI in our hospital or without qualified preoperative images; (3) patients did not undergo spine surgeries or tissue biopsy without pathological findings and (4) patients without complete clinical information (**Figure 1**).

### Image acquisition and data processing

**Figure 2:**
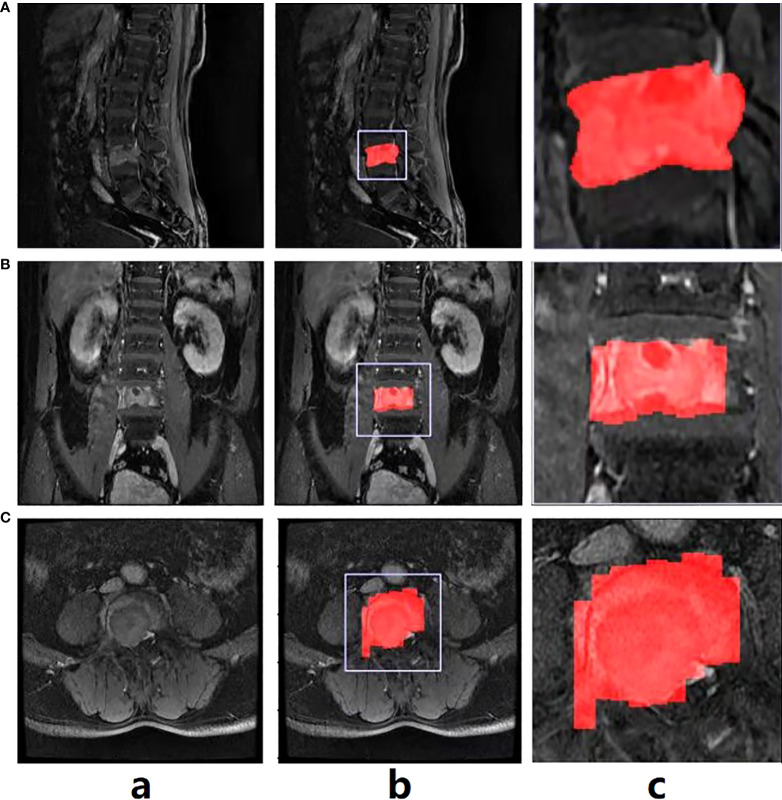
The exemplar spinal image with annotation in the sagittal view **(A)**, and the annotation transfer to the coronal **(B)** and axial views **(C)** using affine transformation.

All patients received MRI examinations using one of three MRI scanners, including 1.5T Siemens, 1.5T General Electric and 3.0T Philips. **Table S1** demonstrates details of imaging parameters from the three scanners. And all scans were performed consistently. Imaging sequences included in this study were CET1 sequences. CET1 was performed after intravenous injection of contrast medium (GD-DTPA Injection, Consun Pharma, Guangzhou, China) according to the recommended dose of 0.2 ml/kg. All sagittal, axial and coronal views were acquired and saved in DICOM format, which was then converted to NIFTI format for the subsequent processes. The tumor lesion’s regions were manually segmented on the sagittal view. The radiologists were blinded to all other information in the process of segmentation, including clinical, imaging, pathological, and follow-up findings. Note that the delineations were firstly done by one musculoskeletal radiologist (5-year experience) using ITK-SNAP software (version 3.8.0), and further checked by another experienced musculoskeletal radiologist (10-year experience). In case of disagreements, re-evaluation would be performed until consensus was reached between the two radiologists. During the assessment phase, the two radiologists made diagnosis independently, who were not informed of the patients’ clinical information and pathology but were told that each lesion were either MM or metastasis. Three-dimensional manual segmentations were performed for entire tumor in all cases. For those with multiple lesions, we manually delineated all the lesions in turn and integrated them into a whole for the subsequent analysis process of radiomics and DL. The tumor diameter was defined as the maximum diameter measured in sagittal MRI sequences. **Figure 2** shows the original CET1 exemplar image of a patient with spinal MM (Column a), with the manual annotations for the tumor region on the sagittal image (**Figure 2A**, Column b). It can be observed that the original normally the tumor region occupies a small part of the whole image (**Figure 2**, Column b). Here we further cropped the image size as 256×256, with the tumor region as the center of the cropped image (**Figure 2**, Column c). In this way, we could unify the size of the training images for model training, and eliminate the background information which is not around the tumor region.

As mentioned above, the manual annotation is made only on the sagittal view (**Figure 2A**, Column b). To conduct the multi-view classification, the affine transformation method was performed in this study. Specifically, DICOM files recorded the position, orientation, pixel spacing and other parameters of each image. The coordinates of any point of the image could be consequently mapped to the human coordinate system. Similarly, given a point in the human coordinate system, the corresponding point in the image could also be found. Therefore, we first converted the coordinates on the sagittal images to the human coordinate system, and then converted them to the coronal (**Figure 2B**, Column b and c) and axial (**Figure 2C**, Column b, c) images. In this way, the annotations in all views could be obtained. Note that due to the spacing between slices during scanning, linear interpolation was used to keep the converted annotations continuous.

The results of alignment are presented in **Figure 2** (Column c). Note that the annotation transfer shown in the figure is not satisfactory, especially in the boundary part which is quite rough. However, it was sufficient as attention guidance for the subsequent classification model training, which could be verified in the experimental section.

### Multi-view attention guided network

**Figure 3:**
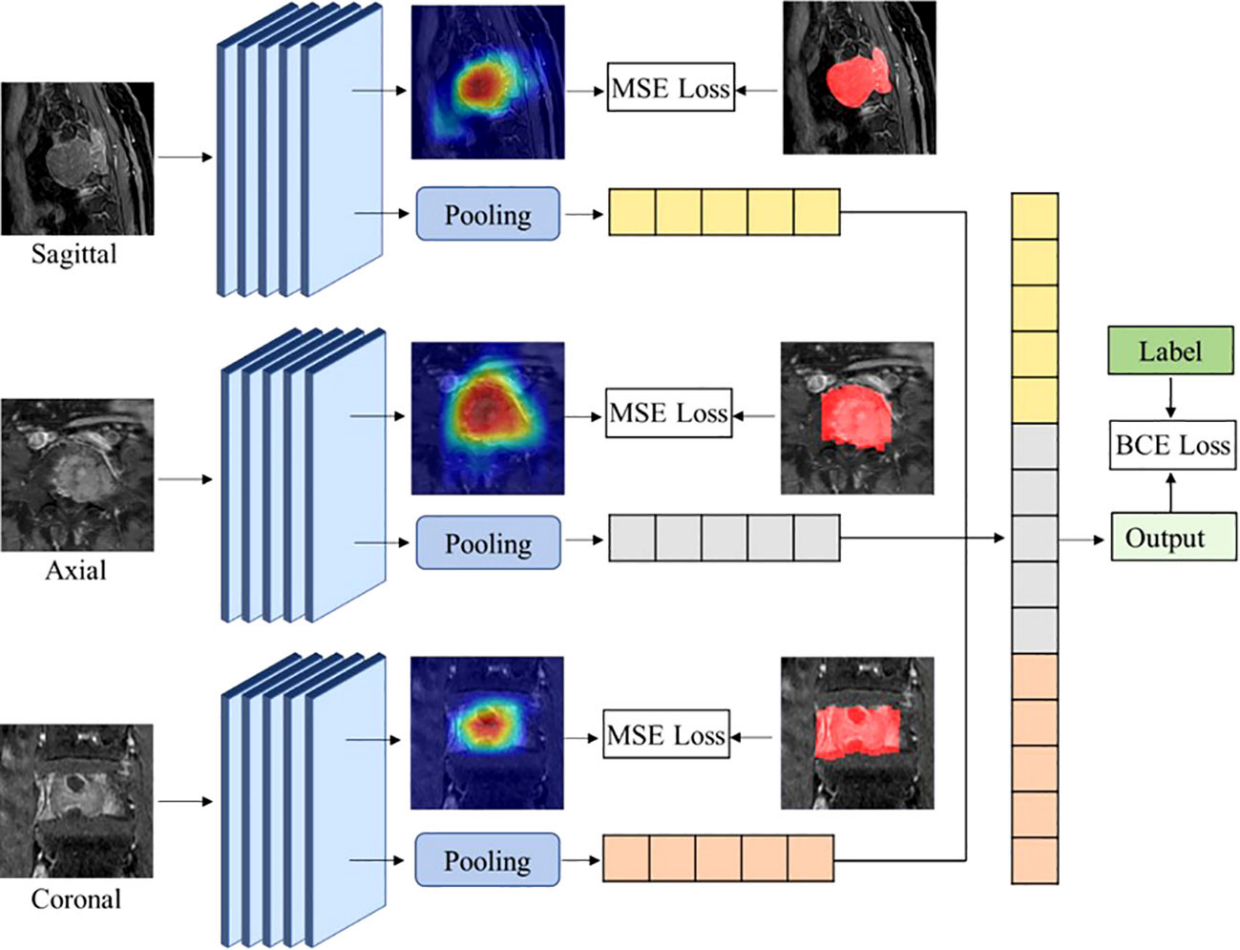
The overall framework for the attention-guided multi-view spinal classification method.

In this study, we designed a novel multi-view attention-guided (MAGN) method for implementing differentiation of spinal MM and metastases, the main framework is illustrated in **Figure 3**. It consists of two major components, which are feature extraction and classification. We first employed the ResNet50 pre-train model as the backbone structure to obtain the visual features from images of three views, respectively. The obtained feature maps were then processed by the LSE (log-sum-exp) pooling layer, and the pooled features from all three views were then concatenated together to obtain the final feature set. Then, the fully connected layer was incorporated with a sigmoid activation, which could output the probability estimate if the subject was spinal MM or metastases.

To ensure the effectiveness of the classification performance, the attention module was also incorporated in the framework, which could obtain the attention map based on the extracted image features using the Class Activation Mapping CAM method (18). Through the back-propagation without updating parameters, CAM could get the importance weights of the convolutional feature maps, and then got the attention map of the network. In this way, we intended to enhance the network’s attention to the lesion region, and integrated extra pixel-level supervision. Specifically, the obtained attention map was then compared with the manually segmented lesion area, which could be further refined and back-propagated to the backbone model to optimize its classification capacity (19).

There were two loss functions designed for network optimization: First, the classification loss function is the binary cross-entropy of classification results and classification labels:


(1) $$\begin{array}{c} L_{C}=-\frac{1}{N}\sum_{I=1}^{N}y_{i}\log\left({\hat{y}}_{i}\right)+\left(1-y_{i}\right)\log\left(1-{\hat{y}}_{i}\right), \end{array}$$

where *y*_*i*_ is the ground truth of the data, ${\hat{y}}_{i}$ is the probability of malignant schwannoma predicted by the model, and *N* is the number of batch size; Second, the segmentation loss function is the mean square error between the network’s attention map and the label map of the tumor region:


(2) $$\begin{array}{c} L_{s}=-\frac{1}{N}\sum_{I=1}^{N}{\left(s_{i}-{\hat{s}}_{i}\right)}^{2}, \end{array}$$

where *s*_*i*_ is the label map and ${\hat{s}}_{i}$ is the attention map. Since we include image information from all three views in this work, the computation of overall segmentation loss *L*_*S*_*all*_ is written as:


(3) $$\begin{array}{c} L_{S\_all}=L_{S\_sagittal}+L_{S\_axial}+L_{S\_coronal}, \end{array}$$

where *L*_*S*_*sagittal*_ , *L*_*S*_*axial*_ and *L*_*S*_*coronal*_ are the segmentation loss for the images in sagittal, axial and coronal views, respectively. The overall loss is therefore written as:


(4) $$\begin{array}{c} L=L_{c}+0.5L_{S\_all}. \end{array}$$

Note that we balanced the priority of the two losses in the computation, and experiments showed that the coefficient shown in the above equation could provide the optimal classification performance. All parameters in the model were first set up following the “He Uniform initializer”^21^, and optimized by Adams optimizer with an initial learning rate of 1e-3, which was reduced by 10% in every 5 epochs. The batch size was set up as 16. The network was built using Python 3.8.3 and Pytorch 1.5. It was trained and tested on an Ubuntu 16.04 computer with GeForce Titan RTX GPU.

### Classification and validation

To validate the MAGN method and radiomics analysis, we conducted 5-fold cross-validation experiments where all subjects were randomly and equally divided into 5 groups following the ratio of spinal MM and metastases. For each fold, one group of subjects was selected as the testing group, while the rest for training. Also note that all models in the experiments were trained using ResNet-50 as the backbone, which was the most suitable pre-train model for this study based on the experiments.

### Differential diagnosis using MAGN and radiomics

The ablation study was conducted to demonstrate the effectiveness of our MAGN method in identifying the spinal MM and metastases. Here we commenced by conducting the differentiation study on the sagittal, axial, coronal views and multi-view separately. In addition, the controlled trials with and without the attention guidance strategy were also designed to clarify their effectiveness. Furthermore, to validate our attention guidance module in visualization, the incorporated Grad-CAM technique (20) was referred to obtain the attention heatmaps for two classification models trained with and without attention guidance.

To further validate the performance of the proposed method, we also conducted a comparative experiment among radiologist assessment, radiomics method and the proposed method. The radiomics features were extracted through the Python package PyRadiomics (21) from the preprocessed images. Radiomics features can be obtained after extraction, which was then utilized for classification through a gradient boosting classifier. The same 5-fold cross-validation was also performed in the experiment using the radiomics method. The performances for different methods of differential diagnosis were all validated by their receiver operating characteristic (ROC) curve and the following three scores: accuracy (ACC), area-under-curve (AUC) and F1-score.

### Statistical methods

Continuous variables of MM and metastases groups were compared through independent samples t-test or Mann—Whitney U-test. Categorical variables were assessed through the chi-square test or Fisher test. Python (version 3.8.1) was used to select features, construct models, and compare the diagnostic efficiency of the models. Clinical data analysis and ROC curve plotting were performed by IBM SPSS (version 21.0, New York, USA). P <0.05 was considered statistically significant.

## Results

### Basic patient information

A total of 217 patients with 269 spinal lesions were enrolled in this study, of which 79 cases were diagnosed as spinal MM with 81 lesions, and the remaining 138 cases were confirmed as metastases from lung cancer with 188 lesions (**Figure 1** and **Table 1**). They aged from 17 to 86 years with a mean of 57.35 ± 12.375 and a median of 59 years with a male-female ratio of 1.4:1. **Table 1** summarizes statistics and comparison of basic information between the two group patients. And there was no significant difference in baseline data.

**Table 1:** Basic clinical information for patients with spinal MM and metastases from lung cancer.

	MM	Metastases	P-value
Age	58.57±11.17	56.64±12.98	0.271
Gender			0.053
Male	53 (24.4%)	74 (34.1%)	
Female	26 (12%)	64 (29.5%)	
Anatomic Site			0.333
Cervical vertebrae	12 (5.5%)	35 (16.1%)	
Thoracic vertebrae	40 (18.4%)	61 (28.1%)	
Lumbar vertebrae	21 (9.7%)	35 (16.1)	
Sacrococcygeal vertebrae	6 (2.8%)	7 (3.2%)	
Tumor Size	4.03±1.17	3.85±1.65	0.379

MM, Multiple myeloma.

### Diagnostic performance using DL methods

**Table 2** shows the comparison classification performance among different DL methods, evaluated by 5-fold cross-validation. It is noted that the multi-view classification achieves the highest scores in ACC (0.79), AUC (0.77) and F1-score (0.69), which also supports our claim that the information from all three views can lead to the improvements in the diagnosis. Besides, it can further improve the performance for all configurations with the incorporation of the attention guidance strategy. Specifically for the multi-view classification method, the ACC, AUC and F1-score have increased to 0.81, 0.78 and 0.71 respectively. Besides, among the three single-view classification models, the model with the sagittal view achieved the best performance with the highest scores of the ACC, AUC and F1-score.

**Table 2:** The Comparison of Classification Performance among Different Methods and Configurations on 5-fold Cross-validation.

Methods	Attention	ACC	AUC	F1-score
Attention guided network				
Sagittal	–	0.7692	0.7546±0.1011	0.6696
	√	0.8061	0.7492±0.0671	0.6893
Axial	–	0.7419	0.7020±0.0268	0.5760
	√	0.7739	0.7196±0.0441	0.6096
Coronal	–	0.7466	0.6642±0.0808	0.5552
	√	0.7834	0.7322±0.1112	0.6694
Multi-view	–	0.7876	0.7661±0.0841	0.6685
	√	**0.8108**	**0.7847±0.1030**	**0.7120**
Radiomics model				
Sagittal	–	0.6035	0.6438 ± 0.0445	0.3474
Axial	**-**	0.6221	0.6417 ± 0.0651	0.3992
Coronal	**-**	0.6682	0.6587 ± 0.0514	0.4616
Multi-View	**-**	0.7098	0.7616 ± 0.0386	0.5363
Radiologist assessment				
Radiologist 1	**-**	0.6544	0.6417	0.5562
Radiologist 2	**-**	0.6912	0.7085	0.6455

ACC, accuracy; AUC, the area under the receiver operating characteristic (ROC) curve.

The bold numbers represent the best result for the deep learning model of MAGN.

**Figure 4:**
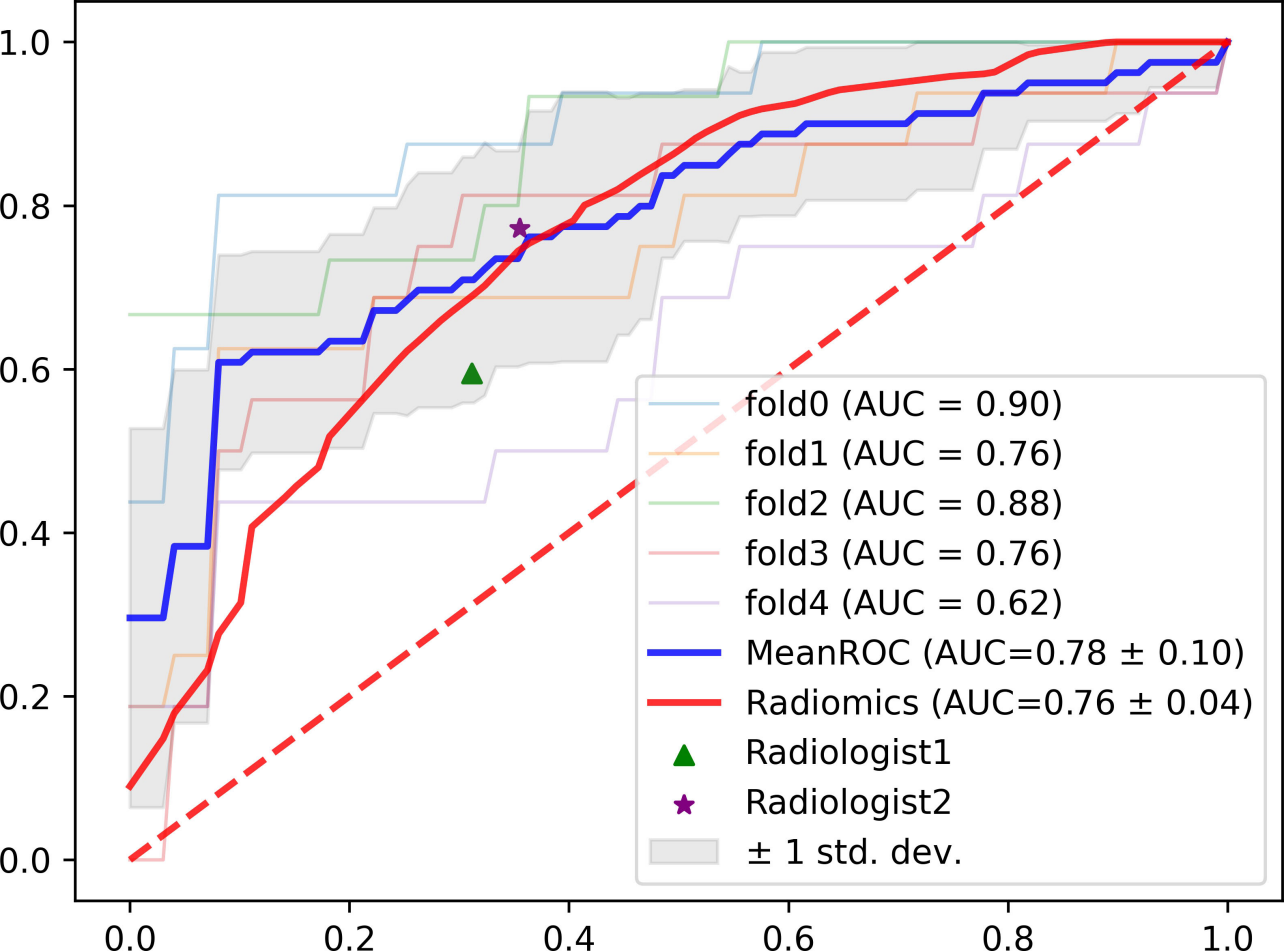
The ROC curves for the five-fold cross validation experiments with the overall performance, radiomics model and radiologist assessment.

Furthermore, **Figure 4** plots the ROC curves representing the classification performances based on the five folds in the AUC score, and the overall performance by averaging the results of all folds. It can be observed that the performance from the five folds is generally stable, and the ROC curve for the overall performance also supports the effectiveness of the trained model.

**Figure 5:**
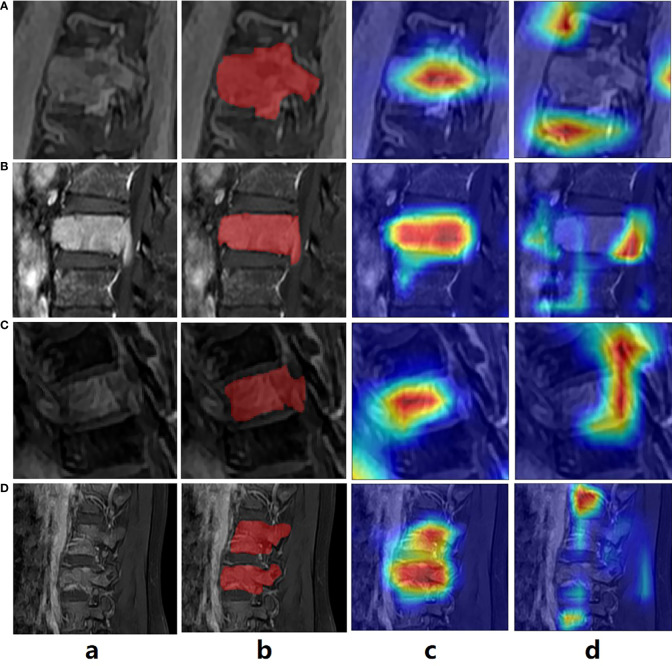
The exemplar of the spinal image with lesion annotation **(A–D)** shows the attention maps generated by the classification model without and with the attention guidance.

To validate our attention guidance module in visualization, which is presented in **Figure 5**. Here, we referred to Grad-CAM technique to obtain the attention heatmaps for two classification models, which were trained with and without attention guidance. It can be observed that the heatmap can better match the lesion region with the help of attention-guided classification (**Figure 5** Column c) than without (**Figure 5** Column d), indicating that our attention-guided classification model is reliable and interpretable.

### Diagnostic performance for radiomics methods and radiologist assessment

In the radiomics model, we acquired 768 radiomics features from the images after feature extraction, including shape, first-order statistics, textural and wavelet features. To further optimize the extracted radiomics features, we further implemented feature selection using the random forest method, which could not only be used for classification but also to rank the priority of the radiomics features. We finally acquired 30 informative features from the preprocessed images, which was then utilized for classification. The specific 30 features selected in sagittal view are shown in **Table S2**. After the ranking of the features was completed, we selected the first half for the final classification. Note that the radiomics feature extraction and selection procedures were applied on three views (axial, coronal and sagittal) of the images independently, which were then used to construct three random forest classifiers, respectively. We also constructed a corresponding multi-view radiomics classifier, where the feature selection was based on the features from all three views. The experimental results are shown in **Table 2**. It can be revealed that the multi-view classifier (ACC 0.71, AUC 0.76, F1-score 0.54) outperforms all of the single view classifier by a large margin, indicating that the multi-view strategy also works for radiomics analysis.

As presented in **Table 2**, the ACC, AUC and F1-score for radiologist assessment were 0.65, 0.64 & 0.56 (Radiologist 1 with 5-year experience), and 0.69, 0.71 & 0.65 (Radiologist 2 with 5-year experience), respectively.

### Comparison among the MAGN, radiomics and radiologist assessment

According to **Table 2** and **Figure 4**, it can be concluded that our proposed method still outperforms radiomics methods and radiologist assessment in differentiation performance with higher ACC, AUC and F1-score (0.81,0.78 and 0.71 of the MAGN vs. 0.71, 0.76 and 0.54 of multi-view radiomics method vs. 0.69, 0.71 & 0.65 of radiologist assessment with 10-year experience). For the three single-view models, the proposed method also conducts higher performance than the radiomics. We attribute them that our MAGN method can extract more comprehensive features that represent the characteristics of the spinal diseases, which help them in better differentiating the spinal MM and metastases.

## Discussion

In this study, we intend to differentiate the spinal MM and metastases originating from lung cancer using the DL model and the radiomics analysis. Specifically, we design a novel Multi-view Attention Guided Network (MAGN) which has shown a stronger capability in differential diagnosis than that of the radiomics model and radiologist assessment. MAGN achieved the highest ACC, AUC and F1-score among all models, trained by CET1 images. Moreover, the incorporation of the attention guidance strategy in MAGN can further improve the efficiency of the model. To the best of our knowledge, this study is the first to differentiate spinal MM and metastases coming from lung cancer by methods of DL and radiomics.

Early diagnosis and differential diagnosis are of great importance in the individualized management for patients with spinal MM and metastases. Histopathological examination of bone biopsy remains the gold standard for detecting benign and malignant tumors, which plays an irreplaceable role in directing the treatment regimen. But its limitations are not negligible due to invasive procedures and limited number of sample sizes. In many cases, pathological diagnose still needs to be performed in combination with imaging signs. However, it is the similar clinical manifestations and radiological imaging finds of the two diseases that make the differential diagnosis extremely difficult by conventional imaging examination including X-ray, CT and MRI (5, 11, 11, 22). Some advanced MRI technologies have been proposed to differentiate spinal MM from metastases in several studies, including DEC and DWI. When detecting malignant lesions of bone, DWI has shown benefits in providing a better signal to background ratio (23). Park et al. evaluated the performance of DWI MRI in differential diagnosis between spinal MM and metastases, and suggested that the combination of standard MR and axial DWI had higher accuracy, sensitivity, and specificity than that of conventional MRI (11). Hyejung Hwang et al. also revealed that conventional MRI in combination with DWI could be useful to discriminate between bone plasmacytoma and bone metastasis in the extremities (24). However, DWI still has some limitations, such as poor performance in anatomical resolution, and assessment of fracture risk for osteolytic lesions. Previous studies have demonstrated that DEC-MRI could supply additional information by measuring vascular parameters in spinal tumors. Ning Lang et al. first used the DCE MRI in detecting spinal MM and metastases, and found that myeloma and metastatic cancers have significantly different DCE kinetics using the evaluation of curve patterns, heuristic analysis of DCE characteristic parameters or a more sophisticated pharmacokinetic modeling analysis (12). Although 18F-FDG PET/CT is recommended as the optimal imaging modality in detecting MM, Zhang Jiahui et al. pointed out that vascular parameters measured by DCE-MRI and glucose metabolism measured by 18F-FDG PET/CT from the most aggressive tumor area did not show a significant correlation (1, 25). In addition, DCE MRI is much cheaper and easily accessible with a much lower radiation exposure. CET1 sequences, rather than conventional sequences were therefore selected for analysis to provide more comprehensive information of spinal malignant tumors involved in our study.

Compared with current mainstream imaging methods based on visual assessment, machine learning has provided an objective tool in many clinic tasks such as differential diagnosis, assessment of therapeutic response and prognosis. In the field of spinal tumor, radiomics shows its effectiveness in distinguishing spinal primary or metastatic tumor (6, 26, 27), prediction of gene mutation (28, 29), early reoccurrence (27), condition monitoring (30) and assessment of treatment response (31, 32). However, there are limited studies focusing on the differential diagnosis between spinal MM and metastases. Xing Xiong et al. (5) established radiomics models based on T1WI and T2WI images, which were successfully applied in differentiating spinal MM from metastases for the first time. The accuracy, sensitivity, and specificity of their optimal model reached 0.815, 0.879, and 0.790, respectively. Jianfang Liu et al. also constructed radiomics model based on conventional vertebral MRI data (T1WI and FS-T2WI) to detecting spinal MM from metastases (15). Different from Xing Xiong et al. study, they improved the methods of features selection, and radiomics model established with 10 events per independent variable (EPV) achieved the best diagnostic performance (AUC = 0.84). In this study, we also performed radiomics analysis based on MRI sequences in differential diagnosis between spinal MM and metastases. Unlike earlier studies, our models were constructed based on CET1 sequences, considering that contrast-enhanced imaging may help provide additional information about the spinal malignancies described above. In addition, three-dimensional manual segmentations were performed for all lesions to delineate the entire tumor, which differed from prior studies where volume of interest (VOI) were defined along the largest cross-sectional area on the sagittal or only the largest lesion was selected and delineated for analysis. All these procedures were designed to provide more comprehensive information for assessment of spinal malignancies, which could contribute more to find features differences between spinal MM and metastases. More importantly, all patients with spinal metastases in our study were from a single origin of lung cancer, which contrasted with prior studies including various metastases. It has been well established that extensive heterogeneity is identified between individual cancers (33). Spinal metastases from different origins were all divided into one group in prior studies, which could not guarantee the grouping of biologically homogeneous cancers. In addition, Ning Lang et al. have revealed that there existed significant differences in imaging features between spinal metastases from lung and other cancers (6). Notably, the radiomics based on multi-view in our study had only moderate efficiency in differential diagnosis with the highest ACC of 0.71 and the highest AUC of 0.76 (**Table 2**), which was consistent with the prior two studies. The diagnostic accuracy might be limited by the vascular bone marrow and complicated anatomic structures in the spine.

DL technology has been reported to outperform radiomics in detecting efficiency (6, 34). Despite its widespread use in evaluating other types of cancer, there have been several reports focusing on spinal diseases. Moreover, no study to date has compared the efficiency of DL and radiomics in detecting spinal MM from metastases. In this study, we proposed a novel DL model of MAGN, which outperformed the radiomics method in distinguishing spinal MM from metastases coming from lung cancer with higher ACC, AUC and F1-score (0.81,0.78 and 0.71of the MAGN vs. 0.71, 0.76 and 0.54 of multi-view radiomics method). Meanwhile, the efficiency of MAGA was further improved by the multi-view feature extraction and fusion strategy, which could conduct the differentiation task in a more comprehensive manner. Besides, we incorporated the attention guidance strategy, which could not only provide prior guidance to aid the model training process, but also produced the attention heatmap (**Figure 5**). The heatmap can resolve the interpretability issue in the DL model, which may indicate the most invasive regions of spine and assist radiologists in differential diagnosis.

There have several limitations in this study. Firstly, it is a retrospective study in a single center with a relatively small population. Further multicenter studies need to be carried out to confirm our results. Secondly, we only selected the spinal metastases coming from lung cancer as the group of metastases to be compared with spinal MM. In next research, we will investigate differential diagnosis between MM and other metastases, as well as among spinal metastases from different origins.

## Conclusion

Our findings reveal that the DL model of MAGN can achieve satisfactory performance in differentiating spinal MM from metastases originating from lung cancer, outperforming the radiomics model and radiologist assessment. Our constructed model may become a promising tool for reaching an early diagnosis and optimizing precision medicine in patients suspected of spinal MM or metastases.

## Data availability statement

The original contributions presented in the study are included in the article/**Supplementary Material**. Further inquiries can be directed to the corresponding authors.

## Ethics statement

Our study was approved by the ethics committee of Changzheng hospital for retrospective analysis and did not require informed consent.

## Author contributions

KC, JC, XinZ, and XW contributed equally to this work. KC, JC, XW and QL conducted data acquisition, collection and interpretation. KC made major contributions in manuscript drafting. XinZ, XiaZ and LZ made important contributions to the model designing and building. SC and PW made of image preprocessing and model interpretation. TL and JD made contribution to the manuscript editing. SL made contribute in the data acquisition and annotation. LZ had final responsibility for the decision to submit for publication.

## Funding

This study was funded by the National Natural Science Foundation of China (Grant number 82001812;62001292), Pyramid Talent Project of Shanghai Changzheng Hospital, The key project of the National Natural Science Foundation of China (grant number 81930049); Shanghai Science and Technology Commission (grant number 19411951300).

## Conflict of interest

The authors declare that the research was conducted in the absence of any commercial or financial relationships that could be construed as a potential conflict of interest.

## Publisher’s note

All claims expressed in this article are solely those of the authors and do not necessarily represent those of their affiliated organizations, or those of the publisher, the editors and the reviewers. Any product that may be evaluated in this article, or claim that may be made by its manufacturer, is not guaranteed or endorsed by the publisher.
