# Supplementary material for: Differentiation between spinal multiple myeloma and metastases originated from lung using multi-view attention-guided network
Source: Front Oncol. 2022 Sep 8;12:981769. doi: 10.3389/fonc.2022.981769 (PMC9495278; doi:10.3389/fonc.2022.981769)
Supplement: Supplementary file 1 [file Table_1.docx]

**Table S1 Image acquisition parameters of three MRI scanners.**

| Scanner Model | | General Electric  Signa 1.5T | Philips  Achieva 3.0T | Siemens  Avanto 1.5T |
| --- | --- | --- | --- | --- |
| T2WI | Repetition time | 3500ms | 4500ms | 2500ms |
|  | Echo time | 101ms | 120ms | 94ms |
|  | Slice thickness | 4mm | 5mm | 6mm |
|  | Matrix | 320×224 | 352×160 | 512×512 |
|  | Field of view | 300×300mm^2^ | 200×300mm^2^ | 240×240 mm^2^ |
| CET1 | Repetition time | 500ms | 650ms | 500ms |
|  | Echo time | 14ms | 12ms | 14ms |
|  | Slice thickness | 5mm | 5mm | 3mm |
|  | Matrix | 512×512 | 352×160 | 512×512 |
|  | Field of view | 240×240mm^2^ | 380×380mm^2^ | 240×240mm^2^ |
