# Supplementary material for: Differentiation between spinal multiple myeloma and metastases originated from lung using multi-view attention-guided network
Source: Front Oncol. 2022 Sep 8;12:981769. doi: 10.3389/fonc.2022.981769 (PMC9495278; doi:10.3389/fonc.2022.981769)
Supplement: Supplementary file 2 [file Table_2.docx]

**Table S2 The features of sagittal view used in Radiomics method.**

|  | Features |  | Features | |
| --- | --- | --- | --- | --- |
| 1 | shape2D_Sphericity | 16 | firstorder_90Percentile |  |
| 2 | firstorder_Maximum | 17 | firstorder_Skewness |  |
| 3 | firstorder_MeanAbsoluteDeviation | 18 | firstorder_Kurtosis |  |
| 4 | LongRunHighGrayLevelEmphasis | 19 | firstorder_Skewness |  |
| 5 | HighGrayLevelZoneEmphasis | 20 | firstorder_Variance |  |
| 6 | firstorder_Mean | 21 | GrayLevelNonUniformityNormalized |  |
| 7 | firstorder_90Percentile | 22 | DependenceVariance |  |
| 8 | firstorder_Maximum | 23 | GrayLevelVariance |  |
| 9 | firstorder_Skewness | 24 | LargeDependenceHighGrayLevelEmphasis |  |
| 10 | SizeZoneNonUniformity | 25 | exponential_firstorder_Energy |  |
| 11 | LargeDependenceHighGrayLevelEmphasis | 26 | exponential_firstorder_TotalEnergy |  |
| 12 | firstorder_Entropy | 27 | GrayLevelNonUniformityNormalized |  |
| 13 | firstorder_Skewness | 28 | LongRunLowGrayLevelEmphasis |  |
| 14 | SizeZoneNonUniformity | 29 | DependenceNonUniformityNormalized |  |
| 15 | GrayLevelNonUniformity | 30 | DependenceVariance |  |
